# Evaluation of Botanicals for Management of Piercing–Sucking Pests and the Effect on Beneficial Arthropod Populations in Tea Trees *Camellia sinensis* (L.) O. Kuntze (Theaceae)

**DOI:** 10.1093/jisesa/ieaa101

**Published:** 2020-11-19

**Authors:** Yueyue Tian, Zejun Chen, Xiaoqin Huang, Lixia Zhang, Zhengqun Zhang

**Affiliations:** 1 College of Horticulture Science and Engineering, Shandong Agricultural University, Tai’an, Shandong, People’s Republic of China; 2 Binzhou University, Binzhou, China

**Keywords:** botanical insecticides, tea pests, matrine, pyrethrin, parasitoids

## Abstract

The tea green leafhopper *Empoasca onukii* Matsuda (Hemiptera: Cicadellidae), the orange spiny whitefly, *Aleurocanthus spiniferus* (Quaintanca) (Hemiptera: Aleyrodidae), and the green plant bugs *Apolygus lucorum* Meyer-Dür (Hemiptera: Miridae) are the important piercing–sucking herbivores in tea trees *Camellia sinensis* (L.) O. Kuntze (Theaceae). The goal of this study was to evaluate the laboratory toxicities and field control efficacies of botanical insecticides including matrine, azadirachtin, veratrine, and pyrethrin to three tea pests. Via leaf-dip bioassay, toxicity tests with botanical insecticides indicated that there were significant differences between the LC_50_ values for botanical insecticides within the same insect species. Matrine had the highest toxicity to *E. onukii*, *A. spiniferus*, and *A. lucorum* with the LC_50_ values of 2.35, 13.10, and 44.88 mg/liter, respectively. Field tests showed that, among four botanical insecticides, matrine at dose of 9 g a.i. ha^−1^ can significantly reduce the numbers of *E. onukii* and *A. spiniferus* and the infestation of *A. lucorum* on the tea plants. Furthermore, botanical insecticides matrine and azadirachtin had no obvious influence on the coccinellids, spiders, and parasitoids densities in tea plantations. The results of this study indicated that use of botanical insecticides, such as matrine, has the potential to manipulate the population of *E. onukii*, *A. spiniferus*, and *A. lucorum* and will be an effective and environmentally compatible strategy for the control of tea pests.

Tea plant (*Camellia sinensis* (L.) O. Kuntze (Theaceae)) is a major and intensively managed perennial plantation crop that is grown worldwide to produce nonalcoholic beverage (Zhang and Chen 2015, Tian et al. 2018). The tea green leafhopper *Empoasca onukii* Matsuda (Hemiptera: Cicadellidae), the orange spiny whitefly, *Aleurocanthus spiniferus* (Quaintanca) (Hemiptera: Aleyrodidae), and the green plant bugs *Apolygus lucorum* Meyer-Dür (Hemiptera: Miridae) are the most common and economically important piercing–sucking herbivores of tea plants in northern China (Zhang et al. 2017a). *Empoasca onukii* causes tender tea shoots to wilt and become stunted and scorched (Qiao et al. 2015, Zhang et al. 2017a). The feeding of *A. lucorum* causes small reddish-brown dead spots on the younger tea buds and irregular holes in the tea leaves (Tian et al. 2019). The leaves and branches of tea trees infested with *A. spiniferus* are usually covered with sooty mould, which causes serious reduction in the vigor and production of tea trees (Van Den Berg et al. 2000, Fu and Han 2007). The feeding by adults and nymphs of *E. onukii*, *A. spiniferus*, and *A. lucorum* results in large economic losses and decline in tea quality. In tea plantations of northern China, *E. onukii*, *A. spiniferus*, and *A. lucorum* reproduce 9–11, 4–5, and 5–6 overlapping generations, respectively, and cause estimated tea yield losses ranging from 15 to 50% of the total tea tender shoots, depending on the population densities of the insects (Zhang and Tan 2004, Zhang et al. 2017a, Liu et al. 2019).

Recently, chemical control is usually used for management of tea pests by Chinese tea growers and relies mainly on synthetic chemical insecticides such as pyrethroids and neonicotinoids (Wei et al. 2015). Nevertheless, high frequency of spraying pesticides causes some serious problems for tea industry. More frequent applications of chemical pesticides promote the development of insecticide resistance in the pest population. For example, *E. vitis* populations had developed high resistance to the commonly used pesticides, including bifenthrin, acetamiprid, and imidacloprid (Wei et al. 2015, Zhang et al. 2017b). Some pesticides such as neonicotinoids can negatively affect nontarget beneficial arthropods such as predators, parasites, and pollinators in the tea agroecosystem (Guedes et al. 2015, Potts et al. 2016, Tsvetkov et al. 2017, Liu et al. 2019). Unreasonable chemical control, especially the use of highly water-soluble pesticides, causes some serious marketing problems due to the presence of pesticide residues in commercial tea (Zhao et al. 2018).

Botanical insecticides, in the form of isolated compounds or mixtures of chemicals, exhibit a wide range of biological activities in the elimination of insects, namely toxicants, feeding deterrents, growth retardants, and repellents (Isman 2006, Hikal et al. 2017). Botanical insecticides are highly effective, readily biodegradable, less risk of pest resistance development, and economically cheap in production (Isman and Grieneisen 2014, Khan et al. 2017, Campos et al. 2018, Jaleel et al. 2020). Botanical insecticides are more safe and ecologically acceptable and have less impact on natural enemies and other nontarget beneficial arthropods such as pollinators (Isman 2006, Potts et al. 2016). Therefore, application of botanical insecticides is a reliable control method against a large number of important agricultural insect species in the IPM program (Hikal et al. 2017, Jaleel et al. 2020).

The objective of our study was to evaluate the toxicities of four botanical insecticides including matrine, azadirachtin, veratrine, and pyrethrin against *E. onukii*, *A. spiniferus*, and *A. lucorum* and the efficacies of the botanical pesticides to tea insect pests in tea fields. Overall, our aims are to supply accurate information to tea growers for the rational application of botanical insecticides for control of tea pests and to reduce chemical pesticide use in the Chinese tea plantations.

## Materials and Methods

### Test Insects

*Empoasca onukii*, *A. spiniferus*, and *A. lucorum* adults were obtained from the established laboratory colonies collected from the tea experimental plantation of Shandong Agricultural University in Tai’an, Shandong Province, China using sweep nets. After aspirating from the sweep nets, the insects were reared on the tea cultivar Fuding in ventilated cages (50 × 50 × 50 cm). The colonies of *E. onukii*, *A. spiniferus*, and *A. lucorum* for use in toxicity determination were established under constant laboratory conditions of 25 ± 2°C, 70 ± 5% RH, and a photoperiod of 14:10 (L:D) h (Wang et al. 2017).

### Botanical Insecticides

Four formulations of botanical insecticides were used in this study: matrine (1.3% aqueous solutions [AS], Weiye Biotechnology Co., Ltd., Tianjin, China), azadirachtin (0.3% emulsifiable concentrate [EC], Greengold Biotechnology Co., Ltd., Chengdu, China), veratrine (0.5% soluble liquid [SL], FJ Bio. Co., Ltd., Xi’an, China), and pyrethrin (1.5% water emulsion [EW], KINGBO Biotechnology Co., Ltd., Neimenggu, China).

### Toxicity Determination of Botanical Insecticides to *E. onukii*, *A. spiniferus*, and *A. lucorum*

The leaf-dip method was adopted for the ingestion bioassay to evaluate the toxicities of botanical insecticides against adults of *E. onukii*, *A. spiniferus*, and *A. lucorum*. Five to eight concentrations within a mortality range of 0–100% based on preliminary assays were prepared from serial dilutions using distilled water. Firstly, the tea shoots with two leaves and a bud collected from the insecticide-free tea plantations were individually dipped into one of the solutions for 5 s, removed, and dried on filter paper for 30 min at room temperature. One tea shoot was put into a glass tube (2.5 cm diameter, 20 cm high) and the stem of tea shoot was wrapped with water-wetted degreasing cotton to maintain moisture. Tea shoots were dipped into the distilled water as an untreated control. Five active insect adults were introduced into each tube, and the tube was then sealed by one layer of gauze. Three replications with a total of 30 individuals per concentration were conducted in each botanical insecticide treatment. After the treatment, the glass tubes were kept in an upright position under the laboratory conditions of 25 ± 2°C, 70 ± 5% RH, and a photoperiod of 14:10 (L:D) h. Mortality was recorded after 48 h.

### Efficacies of Botanical Insecticides Against *E. onukii*, *A. spiniferus*, and *A. lucorum*

The field experiments for control efficacy of botanicals against *E. onukii*, *A. spiniferus*, and *A. lucorum* were conducted in two commercial tea plantations consisted of 7- or 8-yr-old tea trees (cv. Fuding) in Rizhao (experiment site 1: 35.207°N, 119.246°E), Shandong Province, China on 20 June 2018 and Tai’an (experiment site 2: 36.221°N, 116.943°E), Shandong Province, China on 21 August 2019, respectively. All experimental plots were received no other spray and subjected to routine farm management. Three doses of each botanical insecticide at 3, 6, and 9 g a.i. ha^−1^ were prepared by dissolving insecticide formulations with water according to the recommended dosage of the pesticide (http://www.chinapesticide.org.cn/hysj/index.jhtml). The temperature ranged between 18 and 22°C. A blank control was used with water. Multiple 10 × 10 m plots (containing 1,200–1,400 tea plants) were established, and each botanical insecticide at each concentration was sprayed in a total of four tea plots as separate replicates. All treatments were applied by the universal MATABI-16 knapsack hand sprayer with a volume of 675 liters ha^−1^. The number of *E. onukii* and *A. spiniferus* was counted in 100 randomly selected third leaves of tea shoots per plant in each plot. The number of tea leaves exhibiting *A. lucorum* damage was counted in 100 randomly selected tea leaves per plot, and the percentage of damaged leaves was assessed. Counts of *E. onukii*-, *A. spiniferus*-, and the *A. lucorum*-damaged tea leaves in each plot were made at the day when spraying botanical insecticides and at the fifth day after spraying. Sampling was conducted no later than 8:30 a.m. because *E. onukii* and *A. spiniferus* are highly mobile and may quickly hide in tea bushes when disturbed or exposed to sun (Zhang et al. 2014a).

The control efficacy of each botanical insecticide treatment against *E. onukii* and *A. spiniferus* was calculated using the equations (Wang et al. 2013):

$$\begin{array}{l} Density\;decline\;(D)\;(\%)=(NI_{0}-NI_{1})/NI_{0}\times 100, \end{array}$$

$$\begin{array}{l} Relative\;control\;ef\;f\;icacy\;(E)\;(\%)=\;(D_{T}-D_{CK})/(100\;-D_{CK})\times 100, \end{array}$$

where NI_0_ and NI_1_ were mean numbers of insects estimated before and after the botanical insecticide treatments, and *D*_T_ and *D*_CK_ represented density decline of *E. onukii* and *A. spiniferus* in the treatment groups and the untreated group, respectively.

For *A. lucorum*, the percentage of damaged tea leaves was used to calculate the control efficacies of botanical insecticides (Jiang et al. 2015):

$$\begin{array}{l} E(\%)=(PDL_{0}-\;PDL_{1})/PDL_{0}\times 100, \end{array}$$

where PDL_0_ and PDL_1_ were mean percentages of damaged tea leaves estimated before and after the botanical insecticide treatments, respectively.

### Effects of Botanical Insecticide Treatments on Natural Enemies

We inspected each plot for the botanical insecticide treatments for spiders, coccinellids, and parasitoids in order to index the effect of botanical insecticides on the population densities of natural enemies. Subplots of 1 × 1 m were sampled within each plot. The numbers of coccinellids, spiders, and parasitoids in each plot were determined by the knockdown method complemented by visually inspecting plants following Zhang et al. (2014b). Both sampling methods were directed to the upper parts of plants. Knockdown techniques consisted of pulling parts of the tea plants over a rectangular white-colored pan (60 × 35 × 3 cm), after which the tea plant was struck five times and, and the number of dislodged natural enemy was counted. All the natural enemies were identified to species referred to Zhang and Tan (2004). During the sampling process, the numbers of spiders, coccinellids, and parasitoids were determined by both sampling methods.

### Data Analysis

LC_50_ values were estimated by probit regression analysis with SPSS statistical software (version 18.0, SPSS Inc., Chicago, IL). Significant differences in the toxicity of botanical insecticides to the same insect species were based on the absence of overlap in the 95% confidence limits (CLs) of LC_50_ values. Statistically significant mean values of field survey data were compared using the one-way analysis of variance, followed by Tukey’s HSD method (*P* < 0.05). The relative control efficacies of botanical insecticides were arcsine square root transformed prior to analysis, but untransformed data were presented.

## Results

### Toxicities of Botanical Insecticides to *E. onukii*, *A. spiniferus*, and *A. lucorum*

The estimated 48-h LC_50_ value of matrine for *E. onukii* was 2.35 mg/liter, which was significantly different from the LC_50_ values of pyrethrin (10.64 mg/liter), azadirachtin (11.92 mg/liter), and veratrine (24.04 mg/liter) based on the nonoverlapping CL of LC_50_ values. The LC_50_ values of matrine, pyrethrin, veratrine, and azadirachtin for *A. spiniferus* at 48 h were 13.10, 17.28, 17.77, and 22.96 mg/liter, respectively, and no significant differences in susceptibility were observed among the four tested botanical insecticides. Among four tested botanicals, matrine with a 48-h LC_50_ of 44.88 mg/liter was the compound most toxic to *A. lucorum*. The next most toxic botanical was veratrine with a 48-h LC_50_ of 137.32 mg/liter. The 48-h LC_50_ values for pyrethrin and azadirachtin were the lowest with 407.24 and 592.70 mg/liter, respectively (Table 1).

**Table 1.** Forty-eight-hour lethal concentration (LC) (mg/liter) data for the toxicity of botanical insecticides to *E. onukii*, *A. spiniferus*, and *A. lucorum* adults as determined by the leaf-dip method

Insecticide	*E. onukii*			*A. spiniferus*			*A. lucorum*		
	χ ^2^ (df)	Slope (SE)	LC_50_ (mg/liter; 95% CL)	χ ^2^ (df)	Slope (SE)	LC_50_ (mg/liter; 95% CL)	χ ^2^ (df)	Slope (SE)	LC_50_ (mg/liter; 95% CL)
Matrine	1.59 (4)	1.18 (4.56)	2.35 (1.77–3.12)a	4.33 (4)	0.80 (4.11)	13.10 (7.32–23.46)a	1.31 (4)	0.84 (3.61)	44.88 (24.93–80.78)a
Azadirachtin	2.74 (4)	1.08 (3.83)	11.92 (8.59–16.52)bc	1.07 (4)	0.96 (3.69)	22.96 (19.24–27.39)a	2.33 (4)	1.53 (0.75)	592.70 (549.40–639.42)c
Veratrine	3.18 (4)	1.51 (2.92)	24.04 (15.52–37.25)c	1.16 (4)	1.19 (3.52)	17.77 (13.35–23.66)a	2.09 (4)	0.75 (3.39)	137.32 (126.73–148.80)b
Pyrethrin	3.41 (4)	1.20 (3.76)	10.64 (9.35–12.09)b	2.10 (4)	1.54 (3.09)	17.28 (12.71–23.50)a	2.55 (4)	1.34 (1.50)	407.24 (333.28–497.62)c

Different letters indicate significant differences in the toxicity of botanical insecticides to the same insect species based on the absence of overlap in the 95% CL.

### Efficacies of Botanical Insecticides Against *E. onukii*, *A. spiniferus*, and *A. lucorum*

**Fig. 1.**
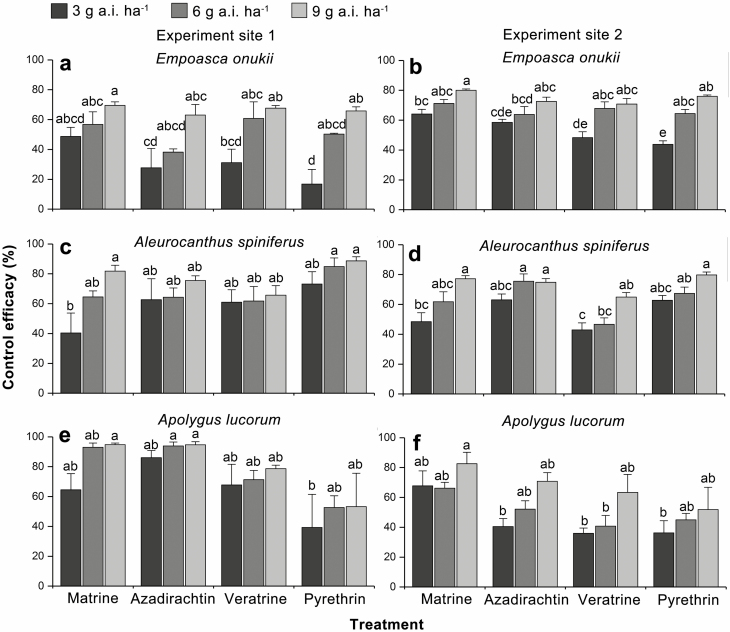
Control efficacies of four botanical insecticides against *E. onukii* (a and b), *A. spiniferus* (c and d), and *A. lucorum* (e and f) in two tea plantations. Values shown are the means and standard errors (±SEs) of four replicates. Different lowercase letters refer to significant differences (Tukey’s HSD test, *P* < 0.05).

The control efficacies of matrine at a dosage of 9 g a.i. ha^−1^ against *E. onukii* were 69.51% and 80.00% in Rizhao (experiment site 1) and Taian (experiment site 2), respectively, which exhibited high efficacy against *E. onukii* compared to other treated groups (Rizhao: *F*_11,47_ = 5.635, *P* < 0.001; Taian: *F*_11,47_ = 11.254, *P* < 0.001). The control efficacies of pyrethrin at a dosage of 9 g a.i. ha^−1^ against *E. onukii* were 65.81% and 76.02% in Rizhao and Taian, respectively (Fig. 1a and b).

In Rizhao, the control efficacies of pyrethrin at the dosage of 9 and 6 g a.i. ha^−1^ against *A. spiniferus* were 88.70% and 84.86%. Nine grams of active ingredient per hectare of matrine showed high control efficacy against *A. spiniferus*, with a control efficacy of 81.81% (*F*_11,47_ = 2.671, *P* = 0.013) (Fig. 1c). In Taian, the control efficacies of pyrethrin at 9 g a.i. ha^−1^, matrine at 9 g a.i. ha^−1^, and azadirachtin at 9 and 6 g a.i. ha^−1^ against *A. spiniferus* were 79.72%, 77.17%, 74.82%, and 75.56%, respectively, and were higher than the control efficacies of other treatments (*F*_11,47_ = 8.568, *P* < 0.001) (Fig. 1d).

The control efficacies of matrine at the dosage of 9 g a.i. ha^−1^ against *A. lucorum* were 94.82% and 82.61% in Rizhao and Taian, respectively (Rizhao: *F*_11,47_ = 3.002, *P* = 0.006; Taian: *F*_11,47_ = 3.637, *P* = 0.002). In addition, in Rizhao, the control efficacies of azadirachtin at 9 and 6 g a.i. ha^−1^ against *A. lucorum* were 94.70% and 93.91%, respectively (Fig. 1e and f).

### Effects of Botanical Insecticide Treatments on Natural Enemies

In tea plantations, the main coccinellid species included *Coccinella septempunctata* Linnaeus (Coleoptera: Coccinellidae), *Harmonia axyridis* (Pallas) (Coleoptera: Coccinellidae), and *Propylea japonica* (Thunberg) (Coleoptera: Coccinellidae). The predominant spider species were *Evarcha albaria* (L. Koch) (Araneae: Saticidae), *Plexippus paykulli* Audouin (Araneae: Saticidae), and *P. setipes* Karsch (Araneae: Saticidae). The main parasitoid species were *Amitus hesperidum* Silvestri (Hymenoptera: Platygastridae), *Apanteles adoxophyesi* Minamikawa (Hymenoptera; Braconidae), *Aphelinus mali* Haldeman (Hymenoptera: Apelinidae), and *Ephedrus plagiator* (Nees) (Hymenoptera: Apelinidae).

**Fig. 2.**
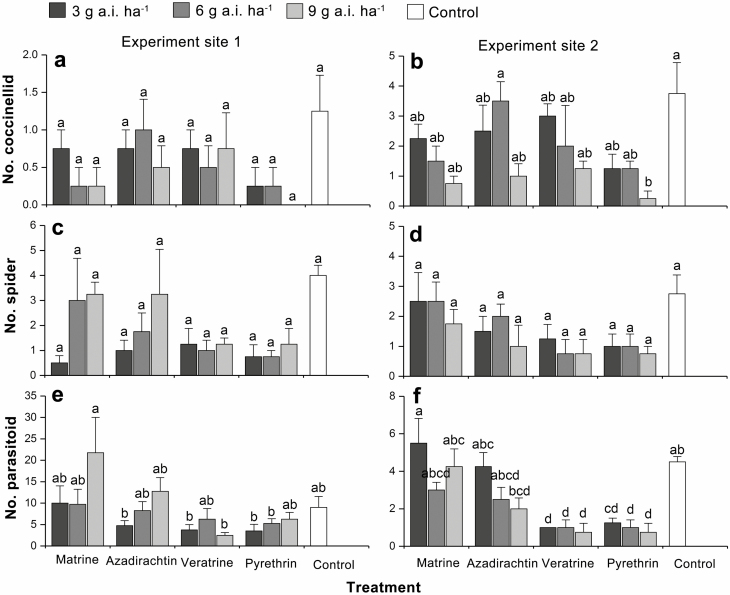
Effects of four botanical insecticides on the numbers of coccinellids (a and b), spiders (c and d), and parasitoids (e and f) in two tea plantations. Values shown are the means and standard errors (±SEs) of four replicates. Different lowercase letters refer to significant differences (Tukey’s HSD test, *P* < 0.05).

In Rizhao, the population densities of coccinellids, spiders, and parasitoids in plots treated with four botanical insecticides were not significantly different than that in the control treatment, respectively (coccinellids: *F*_11,47_ = 1.339, *P* = 0.237; spiders: *F*_11,47_ = 2.090, *P* = 0.041; parasitoids: *F*_11,47_ = 2.538, *P* = 0.014). In Taian, the coccinellid density in the plots treated with pyrethrin at 9 g a.i. ha^−1^ was significantly lower than that in the untreated plots (*F*_11,47_ = 47, *P* = 0.007). There were no significant differences in the spider population densities between the four botanical insecticide treatments and the untreated control (*F*_11,47_ = 7.280, *P* < 0.001). The densities of parasitoids in the plots treated with veratrine and pyrethrin were significantly lower than the densities in the untreated control plots (*F*_11,47_ = 1.745, *P* = 0.094) (Fig. 2).

## Discussion

The key data of our study showed that there were significant differences between the LC_50_ values for four botanical insecticides tested against *E. onukii*, *A. spiniferus*, and *A. lucorum*. The differential toxicity for three different tea pests might be partly attributable to the differences in the mechanism of action of botanical insecticides and the detoxification enzymatic constitution of the species (Zanardi et al. 2015). Matrine had the higher toxicity to *E. onukii*, *A. spiniferus*, and *A. lucorum* than azadirachtin, veratrine, and pyrethrin, and showed potential for pest management of the piercing–sucking herbivores of tea plants owing to its relatively high lethal activities. Wang et al. (2016) and Zhao et al. (2013) demonstrated that matrine and azadirachtin showed high laboratory toxicities against *A. lucorum* and grape leafhopper *Erythroneura apicalis* Nawa (Hemiptera: Cicadellidae) on grape *Vitis vinifera* L. (Vitaceae). Matrine also had high bioactivities against other whitefly species, such as the spiraling whitefly *Aleurodicus dispersus* Russell (Hemiptera: Aleyrodidae) (Lv et al. 2009). Matrine targets acetylcholine (Ach) receptors in insects which in turn effects the production of enzyme acetylcholinestrase (AchE) in the central nervous system of insects (Liu et al. 2008, Senthil Nathan et al. 2008). Ali et al. (2017) reported that matrine treatment resulted in inhibition of AchE activities in sweetpotato whitefly *Bemisia tabaci* (Gennadius) (Hemiptera: Aleyrodidae) and in turnip aphid *Lipaphis erysimi* (Kaltenbach) (Hemiptera: Aphididae).

The results of the field trials further verified that matrine could significantly reduce the numbers of *E. onukii* and *A. spiniferus* and the infestation of *A. lucorum* in tea plantations compared with other tested botanical compounds. Wen et al. (2008) showed that matrine used at a concentration of 7.5 g a.i. ha^−1^ exhibited control efficacy against *E. onukii* was 63.50% in the tea plantations, which was similar to the control efficacies of matrine obtained in our studies. Wang et al. (2016) also demonstrated that the control efficiency of matrine at a dose of 4 g a.i. ha^−1^ against *A. lucorum* on grape *V. vinifera* was obviously higher than the control efficiencies of other four botanical insecticide treatments. Matrine could degrade relatively quickly in the environment with a half-life of approximately 7 d (Xiang et al. 2012). Degradation of botanical insecticides may be attributed to the instability of the active ingredients under ultraviolet light, visible light, and ambient temperature (Turek and Stintzing 2013). Although the low residual persistence might be considered as a disadvantage because of the need to spray botanical insecticides more frequently for effective control of insect pests, this characteristic allows its application preharvest tea shoots and leaves, when it is necessary to use botanical insecticides with a reduced security interval (Zanardi et al. 2015). In addition, the low persistence of botanical insecticides reduces the risks of evolution of resistance in targeted insect pests due to the lower selection pressure (Zanardi et al. 2015).

Although pyrethrin had the lower laboratory toxicity to *A. spiniferus*, pyrethrin also showed higher filed control efficacies against *A. spiniferus*. In organic agriculture, pyrethrins are among the widely used botanical insecticides owing to their high efficacy against homopoteran insect pests including whiteflies and limited persistence in the environment (Prota et al. 2014). Pyrethrin was found to be highly effective in controlling the population of *B. tabaci* on zucchini squash, *Cucurbita pepo* L. (Cucurbitaceae) and poinsettia *Euphorbia pulcherrima* Willdenow (Euphorbiaceae) (Price and Schuster 1991, Razze et al. 2016). The mechanism of action of pyrethrin might be the feeding inhibition effect on whiteflies (Prota et al. 2014).

Botanical insecticides such as matrine and azadirachtin had no influence on the abundance of beneficial arthropods including coccinellids, spiders, and parasitoids in tea fields. Hwang et al. (2009) indicated that plant extract containing matrine and neem exhibited low lethality to predatory and parasitic natural enemies based on the criterion of International Organization of Bio-Control (IOBC). The lower toxicological risk of matrine for these biological control agents might be due to the readily biodegradable characteristic under natural conditions. In the meanwhile, matrine was also low toxic to nontarget organisms, such as pollinators, in the tea ecosystem, and had no carcinogenesis, teratogenesis, and mutagenesis (Ma et al. 2018). However, pyrethrin strongly reduced the population density of parasitoids in tea fields. Previous studies showed that pyrethrin was not absolutely safe to or had been proved highly toxic to natural beneficial parasitoids of pests. For example, Jansen et al. (2010) indicated the potentially high toxicity of pyrethrins for some beneficial arthropod species, such as the parasitic wasp *Aphidius rhopalosiphi* (Destefani-Perez) (Hymenoptera: Braconidae) and the ladybird *Adalia bipunctata* (L.) (Coleoptera: Coccinellidae). Therefore, botanical insecticides such as matrine and azadirachtin might be incorporated into IPM programs in combination with biological control agents for control of *E. onukii*, *A. spiniferus*, and *A. lucorum* in tea plantations.

The results obtained in this study showed that botanical insecticides such as matrine can provide effective control of the piercing–sucking herbivores of tea plants including *E. onukii*, *A. spiniferus*, and *A. lucorum*. Considering that tea pest resistance to chemical insecticides and pesticide residues in commercial tea is an increasing phenomenon (Zhang and Chen 2015, Zhao et al. 2018), use of botanical insecticides may be a promising alternative to chemical control methods due to their control efficiencies against tea pests and their compatibility with natural enemies. This strategy should be integrated with physical controls, biological controls, and some other population-reducing methods to increase the efficacies of botanical insecticides for managing the tea pests in tea plantations.
